# Characterization and Application of Gelatin Films with Pecan Walnut and Shell Extract (*Carya illinoiensis*)

**DOI:** 10.3390/polym12061424

**Published:** 2020-06-26

**Authors:** Juliana Villasante, Anna Martin-Lujano, María Pilar Almajano

**Affiliations:** Chemical Engineering Department, Universitat Politècnica de Catalunya, Av.Diagonal 647, 08028 Barcelona, Spain; julianavillasante@gmail.com (J.V.); anna23martin@gmail.com (A.M.-L.)

**Keywords:** gelatin based films, pecan walnut, shell, antioxidants, physicochemical, meat

## Abstract

Phenolic compounds that come from natural products are a good option for minimizing lipid oxidation. It should be noted that these are not only introduced directly into the food, but also incorporated into edible biofilms. In contact with food, they extend its useful life by avoiding contact with other surface and preventing deterioration air, one of the main objectives. In particular, gelatin is a biopolymer that has a great potential due to its abundance, low cost and good film-forming capacity. The aim of this study has been to design and analyse gelatin films that incorporate bioactive compounds that come from the walnut and a by-product, the walnut shell. The results showed that mechanical and water vapor barrier properties of the developed films varied depending on the concentration of the walnut, shell and synthetic antioxidant. With increasing walnut concentration (15%) the permeability to water vapor (0.414 g·mm/m^2^·day·Pascal, g·mm/m^2^·day·Pa) was significantly lower than the control (5.0368 g·mm/m^2^·day·Pa). Furthermore, in the new films the elongation at the break and Young’s modulus decrease by six times with respect to the control. Films with pure gelatin cannot act as an antioxidant shield to prevent food oxidation, but adding pecan walnut (15% concentration) presents 30% inhibition of the DPPH stable radical. Furthermore, in the DSC, the addition of walnut (15 and 9% concentrations), showed the formation of big crystals; which could improve the thermal stability of gelatin films. The use of new gelatin films has shown good protection against the oxidation of beef patties, increasing the useful lifetime up to nine days, compared to the control (3–4 days), which opens up a big field to the commercialization of meat products with lower quantities of synthetic products.

## 1. Introduction

The food industry has been gaining interest in biodegradable food packaging, not only because they protect the product, but they can also incorporate bioactive compounds that help lengthen the food’s shelf-life. Studies confirm that protein based biodegradable films present excellent mechanical properties and a good barrier holdout for oxygen, microorganisms and humidity [1]. Furthermore, they are nature friendly and safe [2].

The partial acid/base hydrolysis of collagen leads to gelatin production, which comes from bones and skin from different sources such as fish or stock (bovine, porcine and avian) [3]. This protein is of great interest, due to its low cost and easy production. The main disadvantage of gelatin based films is their hydrophilicity and, as a result, high water permeability [4]. Adding hydrophobic compounds would allow to improve this trait. Several studies have shown that adding natural compounds to gelatin based films allows a controlled release of the active components (tocopherols, phenolic compounds and essential oils) and also helps prevent lipid oxidation and microbial contamination [5]. Examples of this are the addition of some compounds such as a curcumin derivative [6], rosmarinic acid, cinnamon bark essential oil [7], green tea, grape seed, ginger, gingko leaf extract [8] and acerola power [9]. The possibilities and studies are very vast. Some have shown very positive results, because there has been an increase in total polyphenols, radical scavenging activity and antimicrobial activity in the foods that it has acted upon. Furthermore, there are other investigations that show that the interaction between the gelatin protein and polyphenols increases their stability and bioactivity [10]. 

The pecan walnut (*Carya illinoinensis*) belongs to the Juglandaceae family. It comes from the south of the United States and the north of Mexico. It is a high nutritional value nut, due to its polyunsaturated fats, such as linoleic acid [11]. It is also a protein, vitamin and mineral source and an excellent source of tocopherols, sterols, carotenoids and aliphatic alcohols [12]. A recent study has proven that using pecan walnut as an antioxidant in sardines prevented their deterioration [13]. Other studies have shown that including the use of pecan walnut in diets can reduce the incidence of cardiovascular diseases, like type 2 diabetes, Alzheimer, Parkinson and cancer [14,15,16]. 

Other parts of the pecan walnut, such as the leaves, the fruit, the green shell and the dry shell reveal a high content in photochemical compounds (phenols, condensed tannins, flavonoids and procyanidins) [17,18,19,20]. The shell of this fruit represents 40–50% of the walnut’s weight [21]. Nowadays, the shell is used for infusions and it is also considered an organic residue from the food industry, which can cause serious problems to the environment [22,23,24].

The aim of this study is the development of gelatin based film for food packaging with the addition of different concentrations of pecan walnut, shell and commercial preservatives, characterizing them and studying how they affect the oxidation of beef burgers when they are brought into contact at a cooling temperature. The addition of this residue aims to improve not only the physical and mechanical properties of films but also to increase the antioxidant activity.

## 2. Materials and Methods

### 2.1. Materials 

All the chemical reagents including gallic acid, 2,2-diphenyl-1-picrylhydrazyl, Trolox, methanol, ethanol, triptone soya agar and Ringer solution were obtained from Sigma-Aldrich (Sigma-Aldrich, St. Louis, MO, USA). Sorbitol was purchased from Calbiochem; Neutral Gelatin from Hacendado (Barcelona, Spain); pure water (Millipore-Q system, Barcelona, Spain). Synthetic antioxidant “Commercial” (dextrin, dextrose, 5.7% SO_2_, potassium metabisulfite, sodium ascorbate and sodium citrate) is commonly used in the meat industry (Barcelona, Spain).

### 2.2. Sample and Extracts Preparation

Pecan walnut (PW) and shell (PWS) were collected in the north of Mexico, they were frozen with liquid nitrogen and shredded with a mortar. Extracts were prepared in order to assay radical scavenging activity and total phenolic content, and a concentrated extract (extract with the ethanol evaporated) to incorporate in the gelatin films. PW and PWS were weighed (3 g) and extracted with 20 mL of 50:50 (*v*/*v*) ethanol:water. The extracts were stirred for 90 min at room temperature. Both extracts were centrifuged and the supernatants were stored at −80 °C. To prepare the concentrated extracts, ethanol was completely evaporated with Rotavapor R-200 with vacuum control V-800.

### 2.3. Film Preparation with PW and PWS Extracts

To prepare the film forming solution, 1.2 g of neutral gelatin and 0.24 g of sorbitol as plasticizer were dissolved in 30 mL of 50:50 ethanol:water solution. Then, the solution was stirred for 10 min at 40 °C until an even mix was formed. Afterwards, different volumes of PW extract were added: 30, 20 and 10 mL (final percentages in the film forming solution 50%, 30% and 10%) that represent 15%, 9% and 3% of initial dry PW, respectively. For the PSW films, only 10% of the extract was used (3% of dry PSW) because higher concentrations were operationally impaired. The solutions were completed up to 60 mL with the ethanolic solution and stirred for 15 min. Then, the solutions were extended in plates (23 cm × 15 cm) and covered with parafilm paper. They were dried at room temperature for 5 days.

### 2.4. Chemical Analysis

#### 2.4.1. Total Polyphenol Content (TPC) of PW and PWS Extract

The Folin–Ciocalteu colorimetric methodology with several modifications was followed in order to determine the TPC with an absorbance in a spectrophotometer [25]. A 1:10 diluted sample with milli-Q water was stirred and the absorbance was measured at 765 nm with Fluostar Omega (BMG Labtech, Ortenberg, Germany). The concentration was expressed as mg of gallic acid equivalents (GAE)/g dry weight (DW) for the total polyphenolic content (TPC). All samples were performed in triplicate. When plotting the absorbance against the gallic acid concentration, the standard curve was achieved, which has a range from 0.11 to 1.72 mM.

#### 2.4.2. Radical Scavenging Activity of PW and PWS

For the radical scavenging activity, DPPH (2,2-diphenyl-1-picrylhydrazyl) method was used [26]. Results are expressed in μmol Trolox Equivalents (TE)/g DW. Measurements were done in triplicate for each sample.

#### 2.4.3. Radical Scavenging Activity of the Films Measured by DPPH

DPPH radical scavenging activity was determined according to the method of Jongjareonrak et al. with some modifications [27]; 0.1 g of the sample was dissolved in 5 mL of methanol for 30 min. After, an aliquot of 20 µL of the methanolic extract was mixed with 200 µL of 0.06 mM DPPH methanol. It was stirred and let to rest at 25 °C in darkness for 75 min. The absorbance of the mix was measured at 517 nm using Fluostar Omega (BMG Labtech, Ortenberg, Germany).

The inhibition of the stable DPPH radical was calculated by using the following equation:

(1) $$\mathrm{Inhibition}\;\left(\%\right)=\frac{A_{0}-A_{S}}{A_{0}}\;\cdot \;100$$

where *A*_0_ is the absorbance of the initial DPPH solution at 517 nm, and *A_S_* is the absorbance of the DPPH solution exposed to the sample at time 75 min.

#### 2.4.4. Fourier Transform Infrared (FTIR) Spectra

The method followed was reported by Staroszczyk et al., with some modifications [28]. FT-IR was the Spectrum Two ™ FT-IR Spectrometer (PerkinElmer, Waltham, MA, USA). The spectra were performed in an infrared region between 4000 cm^−1^ and 400 cm^−1^ (transmittance). Each sample was measured eight times.

#### 2.4.5. Differential Scanning Calorimetry (DSC)

The thermal properties of the films were determined by DSC following the methodology carried out by Ramis [29], using a Thermal Analysis System, Mettler–Toledo DSC30 (Schwerzenbach, Switzerland). The films (10.00 ± 0.25 mg) were put into an aluminum DSC open crucible. All samples were cured in a nitrogen atmosphere with an air flow of 50 mL/min. The studies were performed in a temperature range of 30–250 °C, with a heating rate of 10 °C/min. The fusion enthalpies were calculated with the integration of the calorimetric signal using a straight base line, with the STARe software (Mettler Toledo company, Barcelona, Spain).

### 2.5. Physical Analysis

#### 2.5.1. Film Thickness

The film thickness was measured by a handheld micrometer Mitotuyo, No. 7327 (Kawasaki, Japan) with 0.001 mm accuracy. The film was measured at five random positions. The average of these values was accepted as the film thickness.

#### 2.5.2. Water Vapor Permeability (WVP) 

The permeability values to water vapor were measured as they were by Shiku et al., with some modifications [30]. Permeation cups with silica gel were used; then, the films were cut and put into the discs. The supports (the cups) were introduced in an oven that had a relative humidity of 99% at 25.0 ± 0.1 °C for 24 h. They were weighed before and after introducing them into the drier and the entire procedure was repeated twice. To calculate the permeability to water vapor, the following formula was used:

(2) $$WVP\;\left(\frac{g\cdot mm}{m2\cdot days\;\cdot Pa}\right)=\frac{\left(W_{f}-W_{o}\right)\cdot x}{A\cdot t\cdot \left(P2-P1\right)}$$

*W_f_* is the final weight (g), *W_o_* is the initial weight (g), *x* is the film thickness (mm), *A* is the area of exposed film (m^2^), *t* is the time of incubation (days) and (*P*2 − *P*1>) is the vapor pressure differential across the film (Pascal: Pa).

#### 2.5.3. Scanning Electron Microscopy (SEM)

The morphology of the surface and the transversal section of the samples were observed using the Scanning Electron Microscope (SEM) (Nano Nova 230, FEI, Hillsboro, OR, USA). The lyophilized films were cut into two fragments of 2 cm diameter to place them in the aluminum supports adapted to the machine. A gold coating was applied to increase their conductivity through the sample. The applied accelerating voltage was set on 15.0 kV, with an increase of 500 and 2000× [31]. The experiment was carried out in triplicate.

#### 2.5.4. Crystallinity of Film by X-ray Diffraction (XRD)

The XRD analysis was used to study the structural properties of the films. The instrument used was a dust diffractometer analytical X’Pert PRO MPD (Frenchay, Bristol, UK) with 240 mm of radius. For the transmission method, a configuration of convergent beam was used with a focalized mirror and a transmission geometry with flat samples. The Kα radiation of Cu was used (λ = 1.5418 Å) with a measuring time of 300 s per step and two repetitions were performed. The same configuration was used for the reflexing method, but with a Cu radiation of Kα (λ = 1.5406 Å). Again, the theme assuring time was 100 s per step. In both methods, the same operating power of 45 kV–40 mA was used.

#### 2.5.5. Optical Properties Measurement

The color measurements in films were performed using Datacolor International Colorimeter (Lucerne, Switzerland). They were scanned from 400 nm to 700 nm in order to obtain the reflectance, with a standard light (D65 10 Deg). The luminosity was determined by L*, red–green by a* and yellow–blue by b*. The determinations were carried out in triplicate. The L*, a* and b* parameters were used to calculate total color difference (ΔE) according to the following equation [32].

(3) $$\Delta E=\sqrt{\Delta L^{2}+\Delta a^{2}+\Delta b^{2}}$$

where ΔL*, Δa* and Δb* are the differentials between a sample color parameter and the color parameter of a standard is used as the film background (without any extract).

#### 2.5.6. Mechanical Properties

Ultimate Tensile Strength (UTS), Elongation at Break (EB) and Young’s Modulus (YM) of each film sample were measured with a Zwick/Roell (Ulm, Germany) model ProLine with software testXpert II, according to the legislation ISO 527-1:2012 [33]. Rectangular shapes with dimensions of 57 × 13 mm in width were used. YM (MPa), UTS (MPa) and EB (%) were calculated using the following equations:

(4) $$YM=\frac{\Delta \sigma }{\Delta \varepsilon }$$

(5) $$UTS=\frac{F}{A_{0}}$$

(6) $$EB=\frac{\Delta l}{l_{0}}$$

where σ is the uniaxial force per unit surface and ε the proportional deformation, *F* (N) is the applied force, *A*_0_ is the area of the sample (m²), Δ*l* is the elongation variation between clamps (mm) and *l*_0_ is the initial elongation between clamps (mm).

### 2.6. Evaluation in Beef Patties

#### 2.6.1. Preparation of Beef Patties

Beef meat for the patties was purchased from a local food market in Barcelona, Spain. Beef patties were formulated with 1000 g of different sets of ground beef, each one taken from the round part of different cuts, and was minced three times through 4 mm industrial plates. The meat was mixed aseptically for 3 min with 15 g of salt (1.5% m/m final concentration). Subsequently, the mix was flattened and cut by a round cutter into small hamburger patties, each one around 8 g of weight and for microbiology experiments 10 g. The hamburger patties were placed in plastic trays, one control (sample prepared with no wrapping; meat sample only) and the other beef patties were covered on both sides with the different films treatments: control (only gelatin), 15% PW, 9% PW, 3% PW, 3% PWS and 1% commercial. Then the samples were stored in the dark at 4 ± 1 °C.

#### 2.6.2. TBARS Assay

The TBARS value was determined according to the methodology of Villasante et al. [13] with several modifications; 0.5 g of sample were homogenized for 1 min with 0.5 mL EDTA 0.3% solution and 2.5 mL TBARS reagent and an Ultra-Turrax blender was used (Ika-Werke, GmbH & Co, Staufen, Germany). While the determination of all samples was taking place, all samples were maintained in an ice bath. Afterwards they were filtered with Whatman filters no. 1 and the reaction got started with the insertion of the tubes in a water bath at 95 ± 1 °C for 10 min. After cooling, a UV/VIS microplate reader spectrophotometer Fluostar (Paris, France) was used to read the absorbance of the samples at 531 nm. Values of TBARS were shown as mg of malonaldehyde per kg of sample. The experiment was performed in triplicate.

#### 2.6.3. Microbiological Analysis

Ten grams of each sample was dissolved with 90 mL of ringer solution and placed in a stomacher bag (Stomacher^®^ Lab system Seward, London, UK). Then, they were homogenized for 5 min using a Seward stomacher 400 (Seward Medical UAC House, London, UK). Several dissolutions were prepared and 100 μL of each dissolution was spread on a triptone soya agar coated plate. Afterwards, the coated plates were incubated at 35 ± 1 °C for 48 h. Once the incubation finished, the colony forming units (CFU) were determined and reported as log of colony forming units per gram (log CFU/g).

#### 2.6.4. Determination of Metmyoglobin (MetMb)

The determination of metmyoglobin was carried out according to the procedure described by Ouerfelli et al. [34] with some modifications. One gram of meat patty was homogenized with 5 mL of 0.04 M phosphate buffer (pH 6.8) for 30 s using an Ultra-Turrax (IKA, Staufen, Germany). The mixture was refrigerated at 4 °C for 1 h and centrifuged at 12,000× *g* for 10 min at 4 °C. The absorbance of the upper phase was read at 572, 565, 545 and 525 nm using a UV/VIS microplate reader spectrophotometer Fluostar (Paris, France). 

The percentage of metmyoglobin was determined using the following formula:

(7) $$\mathrm{MetMb}\;\left(\%\right)=\left[2.514\times (A_{572}/A_{525})+0.777\times (A_{565}/A_{525})+\;0.8\times (A_{545}/A_{525})+\;1.098\right]$$

where the absorbances: A_572_, A_525_, A_565_ and A_545_ were at a wavelength of 572, 525, 565 and 545 nm, respectively.

#### 2.6.5. Statistical Analysis

The samples were performed in triplicate. The mean and the standard deviation were obtained from each measurement. The significant differences were found by one-way ANOVA. Means were contrasted using Tukey’s test *p* < 0.05. All statistics were carried out using Minitab-18 (Minitab Ltd., Coventry, UK) for Windows.

## 3. Results and Discussion 

### 3.1. Total Polyphenol Content (TPC) and Radical Scavenging Activity (RSA) of PW and PWS

The total polyphenol content (TPC) and the radical scavenging activity (RSA) of PW and PWS, determined in ethanolic aqueous extracts (50:50), have been found to be tightly correlated. Table 1 shows the results of the TPC and RSA from the PW and PWS extracts. Both the TPC and the RSA are three and five times greater in the shell than in walnut. These are not novel values, since similar results can be found by different authors in the bibliography, where the pecan walnut and shell extracts have been found to have seven times more polyphenol content in the shell (92.5 mg GAE/g sample) than in the fruit (11.9 mg GAE/g sample) [20], all of them originating from different parts of Mexico. The researcher Flores-Córdova et al. obtained similar results working with two different varieties of the pecan walnut, Western and Wichita [35]. All the studies conclude that the values obtained for the shell are several times superior to the ones obtained for the fruit. Nevertheless, the exact values and obtained quantities differ between publications due to the different varieties of walnut studied, the geographic area, the year of harvest, soil, agricultural practices and environmental influences, among others.

### 3.2. RSA of the Films by DPPH (2,2-diphenyl-1-picrylhydrazyl)

Nowadays there is an increased interest in food packaging that offers antioxidant properties to avoid or delay lipid oxidation. Methodologies such as DPPH are used to calculate the delay in the production of the free radicals’ formation and determine the radical scavenging activity [36].

**Figure 1:**
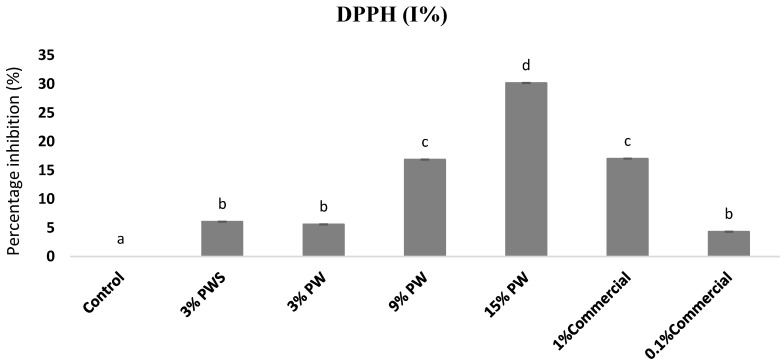
Radical scavenging activity of the films: DPPH (I%). Different letter means are significantly different (*p* < 0.05). PW: Pecan walnut; PWS: Pecan walnut shell.

Figure 1 shows the radical scavenging activity measured by the DPPH methodology in films with different shell and walnut concentrations and also compared to the commercial antioxidant in films. The control, gelatin film without any added extract, has scarce radical scavenging properties due to particular amino acids such as glycine and proline [37].

Data indicated that DPPH^•^ radical-scavenging activity of films increases in a dose-dependent way, incrementing the final content from 3% to 9%, leading to a significant improvement in the radical scavenging activity, which was boosted from about 5 to 30%. Similar results were obtained for the 3% shell concentration, 3% walnut concentration and the 0.1% of the commercial sample which is the concentration usually used by the meat industry. The 9% PW concentration offers a percentage of inhibition similar to the 1% commercial sample. As for the 15% PW concentration, it is more than thirty times greater than the control. The PW extract is comprised in a significant amount of phenolic compounds such as catechin and epicatechin, among others and phenolic acids such as gallic and ellagic acid which have shown antioxidant characteristics [15].

On the other hand, there is a great quantity of tannins present in these extracts and maybe tocopherols in the case of the PW [38,39], although a significant correlation between its concentrations and the radical scavenging activity does not exist. 

Furthermore, various antioxidant compounds can interact with the gelling matrix in different manners, facilitating their release, while Cano et al. found that the EC_50_ (g of tannin/mol DPPH) values obtained for tannin based films were higher than those obtained for the pure tannins (without films). They concluded that the interaction of some phenolics compounds like tannins with proteins such gelatin limited the diffusion of the antioxidant activity [40,41].

### 3.3. Fourier Transform Infrared (FTIR) Spectra

Fourier Transform Infrared spectra were used to determine the types of molecules that were present in the films. Table 2 shows the FTIR spectra of the control, PW, PWS and commercial films. A strong broad band at 3289.11 and 3281.09 cm^−1^ represents the OH and NH groups [42] and was observed in all samples, even though the peak got more intense after the addition of the PW and the commercial preservative. There are two more peaks to be observed between 3007.26 and 3007.59 and also 2934.24 and 2923.27 cm^−1^, which are due to the presence of aliphatic and unsaturated hydrocarbons related to terpenoid compounds [43]. The 3007.26–3007.59 cm^−1^ peak is not found in the control nor in the shell extract, which indicates that it is a compound that can only be found in the walnut, like vitamin E. The second peak 2934.24–2923.27 cm^−1^ is also not found in the shell extract, which could be due to the amorphous cellulose found in the shell [44].

The peak between 1630.86 and 1645.51 cm^−1^ is attributed to a C=O stretch [6], which could represent amide I or an unsaturated ester and carboxylic acid [45]. In the PWS extract, the peak is 36.26% stronger with respect to the control film. The same tendency is observed in the peak between 1548.37 and 1541.41 cm^−1^, which represents the amide II. 

### 3.4. Differential Scanning Calorimetry (DSC) 

**Figure 2:**
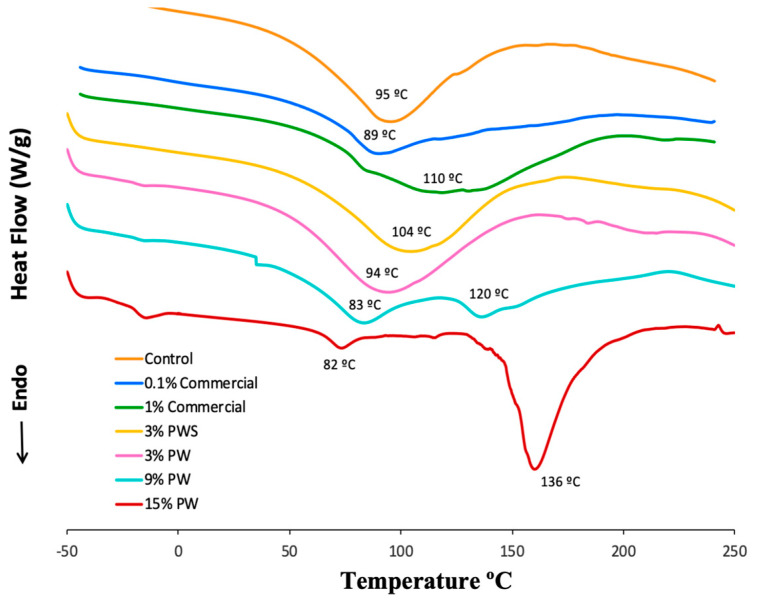
Differential scanning calorimetry (DSC).

The thermodynamic characteristics are useful to determine the quality of films when they are found under different conditions such as temperature, gas pressure or atmospheres (reducing or oxidant). The effect of walnut and shell extracts and the addition of commercial preservatives on the thermal stability of the gelatin films was examined. The samples and control were maintained at equal temperature throughout the analysis. Figure 2 shows the heat flow versus the temperature and several peaks can be observed. The first peaks (89.29–117.51 °C) in all the samples correspond to the natural crystallization of the gelatin. In addition, the transition coil-helix gelatin film facilitates disruption of the molecular ordered structure [46]. The results of fusion transition temperature of the control (94.89 °C) were similar to those obtained by Wang (92.12 °C) [47], who worked with gelatin films incorporating high amylose corn starch. The films with 15 and 9% walnut concentrations present two different peaks, 91.64–133.43 °C and 83.14–135.91 °C respectively. That indicates that they have two points of fusion, which could be due to bigger crystal formation. The samples with 3% PW and PWS concentration do not show significant differences compared to the control, so the fruit quantity and the residue incorporated in the film is not enough to induce the formation of more crystals. The films with commercial preservatives show similar results. 

The addition of PW and PWS could improve the thermal stability of gelatin films. The effect on thermal properties depends on the internal structure. Maybe, PW and PWS contain higher stability, which requires more energy to break the structure, resulting in a higher thermal stability [48].

### 3.5. Physical Analysis

#### 3.5.1. Film Thickness

The film thickness could be correlated with the water vapor permeability and mechanical properties of the film [49]. Table 3 shows the thickness of the gelatin films with the addition of the different extract concentrations. The films obtained showed a thickness range from 0.032 to 0.150 mm. Significant differences (*p* < 0.05) were found between the control and the samples with 15% and 9% PW and 3% PWS concentrations, whereas the ones with added artificial preservatives do not show any significant differences. Given that all the samples were prepared with different percents of extracts that contain different solid components (dry mass), this results in ticker films after evaporation of the solvent. Similar trends were presented for gelatin film containing different apple-peel nanoparticles [48].

#### 3.5.2. Water Vapor Permeability (WVP)

**Figure 3:**
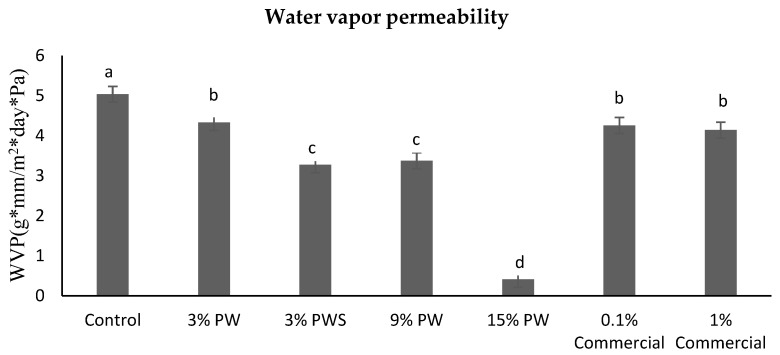
Water vapor permeability (WVP). Different letter means are significantly different (*p* < 0.05). PW: Pecan walnut; PWS: Pecan walnut shell.

Films with gelatin are hydrophilic, so they tend to retain water in their structure, which causes a swelling, showing an insufficient performance. When plasticizing agents such as sorbitol and fatty acids are incorporated, they make the films more permeable to water vapor [50]. Figure 3 shows the water vapor permeability is inversely proportional to the extract concentration. This could be due to the fact that the walnut is rich in monounsaturated fatty acids and tocopherols such as vitamin E [51]. There are hydrophobic compounds that exert a greater barrier to water vapor. In this case, the PWS films present, as expected, a higher permeability to water vapor because it is mainly fiber [21]. The commercial films (both concentrations) show the same behavior as the 3% PW; this could be due to the dextrose and dextrin content in the commercial additive [52].

#### 3.5.3. Scanning Electron Microscopy (SEM) 

**Figure 4:**
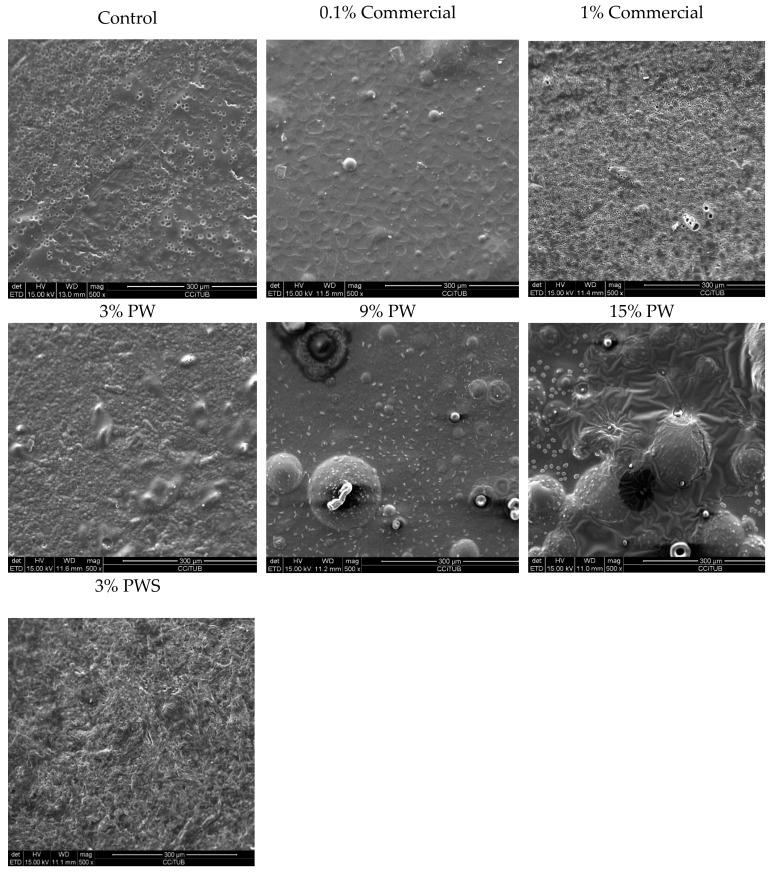
SEM images of the cross-sections or surface of gelatin films with the addition of different concentrations of walnut, shell and commercial preservative. PW: Pecan walnut; PWS: Pecan walnut shell.

Mechanical properties and water vapor permeability could be affected due to the films’ microstructure, like the morphology and homogeneity of the matrix [53]. Figure 4 shows SEM images of the films’ surface. SEM images show that the outer layer of the control film was flat, uniform, ordered and in homogeneous structure without bubbles or pores. It can be observed that, with increasing PW concentration, the matrix is more heterogeneous. This is due to a phase separation, because the gelatin and the PW have a hydrophilic and a hydrophobic nature, respectively. Acosta et al. [54] performed some studies in which they added esters of fatty acids to gelatin and yucca starch films and a phase separation also occurred [54]. Other authors reported a similar phenomenon [55]. It is clear from the SEM analysis, that the films that contained extracts had an altered structure and presented some discontinuities in the matrix (Figure 4). Nano-particles were formed when the ethanolic extract was blended with water due to the fast evaporation of non-water-soluble extract components. Gonçalves et al. studied the hydrophobic properties of some antioxidants, which could lead to the microprecipitation of this hydrophobic unit [56]. Morphological changes in the films such as a rougher surface were observed due to changes in solubility. Similar results were found by Pastor et al. and Chang-Bravo et al. who studied biopolymer films carrying ethanolic extracts of propolis [57,58]. In addition, Riaz et al. also obtained similar results, which show that the samples with the lowest apple peel ethanolic extract have a good dispersion in the film, whereas in the other samples, agglomerates of nanoparticles were described. In fact, a low extract concentration resulted in a more homogeneous particle size distribution [48]. Teodoro et al. studied the fabrication of cassava starch films containing acetylated starch nanoparticles as reinforcement [59].

In this work, the shell presents a more homogenous but roughened disposition. This is concordant with results obtained by Iahnke et al. with the incorporation of beetroot in gelatin films [60] and Harini et al. who studied a film based on walnut shell fiber and cashews [44].

The film with a 0.1% commercial preservative shows a more homogeneous dissolution, whereas the 1% commercial film shows porosity and roughness.

#### 3.5.4. Crystallinity of Film by X-ray Diffraction (XRD)

**Figure 5:**
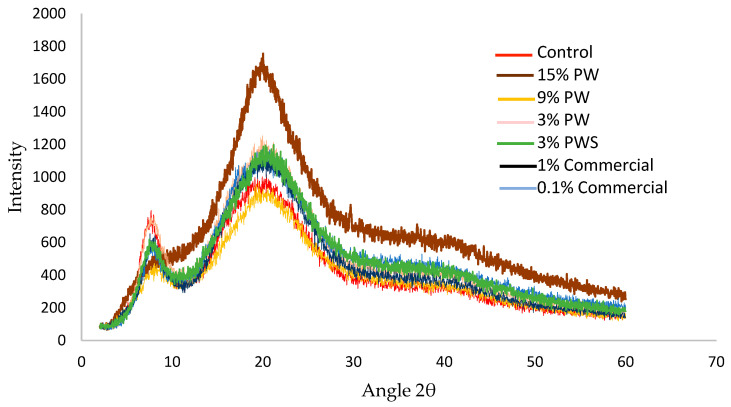
X-ray of gelatin films with the addition of different concentrations of walnut, shell and commercial preservative. PW: Pecan walnut; PWS: Pecan walnut shell.

Depending on the internal distribution that bonds the atoms, solids can be classified as amorphous, polycrystalline or crystalline. This study is performed through X-ray diffraction. Figure 5 shows an acute and dominant peak between 7.5 and 8.2° (2θ) in all samples, which decreases its intensity on increasing the PW or PWS concentration. The other peak is between 18 and 25° (2θ) also in all the samples. These two peaks are found in d = 11.5 and 4.5 Armstrong (Bragg’s Law), respectively [61], which indicated the mean difference between the reticular planes (it is not crystalline, so it cannot be specifically named distance between planes).

These peaks indicate the reconstitution of the collagen-like triple-helix structure of partially crystalline gelatin. The intensity of this peak was assigned to the content of triple helix [62,63].

Similar results were shown by Pępczyńska et al. with salmon gelatin film—a high intensity peak is observed at the diffractive angle 2θ ~9°—that they concluded, similar to Rivero et al., could be associated to the formation of triple helices (in the process of salmon gelatin film formation). In the same study Pępczyńska et al. found a broader diffractive peak in a 2θ diffraction angle range of 11–30° [62,64].

On the other hand, similar results were found for gelatin films with different concentrations of epigallocatechin gallate fabricated by thermo-compression molding added. Nilsuwan et al. found that with a high concentration of this phenolic compound there was a higher peak intensity. They suggested that this result could be for a higher number of interactions between gelatin and the epigallocatechin gallate via hydrogen bonding [65].

#### 3.5.5. Optical Properties Measurement

Color is one of the most important sensory quality attributes for consumers. Usually, protein and polysaccharides-based films are colorless, depending on the concentration and the type of amino acids present and the treatment applied. Uranga et al. worked with anthocyanins. In their study, anthocyanins were applied to gelatin films, obtained in their control (only gelatin) with similar values to the ones shown in Table 4 (L* = 94.86, a* = −0.11 and b * = 4.98) [43].

The addition of PW and PWS in gelatin films caused an increase in the yellowness (b*), in the redness (a*) and the total color difference value (ΔE*) compared to the control. There are no significant differences between the two commercial concentrations, but when the PW and PWS concentration increase, the luminosity (*L) is reduced compared to the control. This is because the PW and PWS is opaque, due to its polyphenolic compounds; the coffee color comes from compounds that the shell and walnut contain, such as tannins [38,66].

#### 3.5.6. Mechanical Properties of the Films 

The food industry is developing new food packaging that is capable of maintaining the food properties and their integrity during the life of the product [67,68,69]. For this reason, the mechanical properties of the packaging such as rigidity, tension and percentage of deformation have a relevant role. The results of mechanical parameters (Young’s modulus, tensile strength and elongation at break) of the analyzed films are presented in Table 5. It can be observed that the control film, 3% PW and PWS and the two different commercial concentrations do not present significant differences regarding the Young’s modulus, UTS and EB. Additionally, they present higher YM and UTS parameters, which means that they present a greater resistance to elastic deformation, so they are more rigid and show a higher deformation tension. Due to this, their percentage of deformation is smaller, whereas for the films with a higher PW concentration, the breaking tension and the Young’s modulus decrease compared with the control, up till six and eight times, respectively. The elastic deformation increased significantly (*p* < 0.05). Results with the same tendency were reported in different studies: Ge et al., who added rosmarin extract to gelatin films [70], Phebe and Ong worked with starch–chitosan films incorporated with oregano essential oil [69], Putsakum et al. studied the properties of gelatin films developed from neem extract [71] and Bonilla et al. studied the effect of eugenol oil and ginger addition in gelatin films [72]. In all these studies, it can be concluded that, when polyphenolic compounds and fatty acids are added, they provoke an interaction with the gelatin matrix (protein), forming hydrogen bridges and covalent bonds with amine and hydroxyl groups and inducing the debilitation of protein–protein interactions [8]. Similar effects have been found in the addition of oxidized linoleic acid compared with the control, presenting a lower elastic modulus and tensile strength (8–42% and 21–57%) but higher elongation at break (366–956%) [73]. This study claims that incorporation of fatty acids such as linoleic acid to films could reduce the intermolecular interaction among gelatin molecules, resulting in increasing chain mobility and free volume in the film matrix. Instead, tannins are the main polyphenolic compound in the pecan walnut. Cano et al. added tannins from oak bark to gelatin-based films; they found that the resistance to break and elastics modulus significantly decreased. They mentioned that this fact could be due to the precipitation of the gelatin–tannin complexes, which is caused by the presence of strong electrostatic connections between the different charged groups (gelatin: Positive; tannins: negative) [40]. On the other side, the incorporation of 0.1 and 1% commercial preservatives did not show significant differences with respect to the control (*p* < 0.05).

Therefore, the incorporation of PW and PWS concentration could affect the mechanical properties of gelatin films differently. It depends on the method used in the extract addition and the concentration incorporated.

#### 3.5.7. Film Protection in Beef Patties

Edible films and coatings reduce lipid oxidation, which preserves the quality of meat products during their storage time [74]. In order to have a higher concentration of antioxidants in a patty’s surface, antioxidant extracts can be added to the formulation of films. Protein films have a tightly packed, organized hydrogen-bonded network structure and therefore are good oxygen barriers. Gelatin films are a type of protein-based polymer, which has been broadly used to form edible films.

Films made from proteins are excellent oxygen barriers because of their tightly packed, ordered hydrogen-bonded network structure. A particular case is gelatin films, a protein-based polymer widely used as a starting material for edible film formation. 

In the previous sections, it has been proven that films (both the ones that contain the walnut shell and the ones that contain the walnut itself) have radical scavenging activity and good physical properties. Therefore, they are potentially useful to protect food against oxidation once in contact with the food itself. To show its antioxidant activity in a real food, an experiment has been designed in which different films are set above and under small beef patties (8 g, 3 cm of diameter). Six small patties are set in any tray; five of them are protected with one of the different films designed and the control sample does not have a film. Each full tray is covered with transparent film suitable for food.

Periodically, one tray is removed to do the analysis on secondary oxidation through the TBAR compounds. TBARs were measured as an indicator of the secondary phase of lipid oxidation as mg malondialdehyde per kg of sample (mg MDA/kg beef). The deterioration of the secondary lipids would alter the flavor and contribute a rancid odor and unfavorable taste to the food [75].

The metmyoglobin as the responsible compound to the loss of red color and the apparition of a brown coloration has also been followed and analyzed.

**Figure 6:**
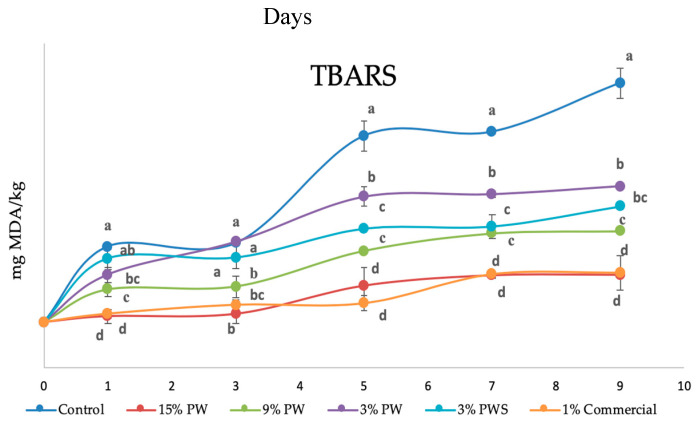
Thiobarbituric acid reactive substance (TBARS) values of raw beef patties during storage at 4 ± 1 °C. results show the mean performed in triplicate (*n* = 3) and are shown as a mean value ± SD. Different letters in a day illustrate significant differences between the samples at *p* < 0.05, whereas different capital letters show significant differences between storage days at *p* < 0.05 for the same sample. PW: Pecan walnut; PWS: Pecan walnut shell.

The results of the evolution in time of the compounds reactive to tiobarbituric acid (expressed in mg of malondialdehyde (MDA)/kg of meat) against time (nine days in total) are summed up in Figure 6.

It is interesting to point out the static situation that appears after seven days; four different types of samples can be distinguished. The oxidized sample (which reaches higher values than 1.2 mg MDA/kg of meat) is, as should be expected, the control, that is, the mini-hamburger that does not have any gelatin film protections (it does have protection with the commercial film, common to all patties).

These TBAR values make the sample unacceptable for its consumption, due to the MDA quantities between 1.0–2.0 mg /kg, which make the product have off-flavors that make it reprehensible [36,76].

The next sample in terms of deterioration (significantly different even with α = 0.01) is the one with the lower walnut quantity (3% PW). It has an oxidation of approximately 0.75 mg MDA/ kg of meat. According to the report by Brychcy [77], rancidity and off-flavor were noticed when the TBAR value was in the range of a 0.5–1.0 mg MDA/kg sample. For this reason, it could be said that the walnut extract concentration within the film is not enough to avoid an oxidation process that allows the meat to be perfectly edible after a week.

The films 3% PWS and 9% PW do not show different behaviors statistically speaking. Both reach values near the limit of sensory acceptability after seven days (values near 0.6 mg MDA/kg of sample). These values are in the same ranges (also considered up to seven days) as the ones obtained by Huiyun Zhang et al. [78], who prepared a natural active chitosan coating loaded with nano-encapsulated *Paulownia tomentosa* essential oils and investigated the effects of the coating treatments in pork chops.

Lastly, there is the group formed by the film with the commercial antioxidant and the film that contains the greater walnut concentration (1% commercial, 15% PW, respectively). In this case the oxidation up to seven days is close to 0.4 mg MDA/kg of meat. This fact shows that the oxidative protection that the film 15% PW transfers is very similar to the one obtained by the commercial antioxidant (1%). Both prevent oxidation, making the beef patty free of off-flavors and rancidity.

Other films, also gelatin ones, where phenolic antioxidants have been added, but tested in a different matrix, such as cod-liver oil, show similar results after 10 days, even though the control is less oxidized (values near a 1 mg MDA/kg sample). However, the protection is maintained longer, up to 20 days, at which time the control, without a film applied, has five times higher oxidation [79].

Bermúdez-Oria et al. also worked with gelatin films and beef meat. The film used pectin–fish gelatin (in this case they came from fish and incorporated pectin) and the specific antioxidants added, hydroxytyrosol and 3,4-dihydroxyphenylglycol [80]. The protection against oxidation (measured through the TBAR formation) was less because the values obtained were between 1 and 2.5 mg MDA/kg meat (compared with the control, which had values near 4 mg MDA/kg meat).

As a conclusion regarding the oxidation, it can be said that films designed with the incorporation of 9 and 15 per cent of PW, like the one with 3% of shell, stabilized the beef patties so that after one week the degree of oxidation made them suitable for consumption.

Regarding the microbial contamination, it has been seen that after a week the log of the CFU/g meat is 4.12 for the control. All samples protected with gelatin films have similar values (expressed in the same units, log of CFU/g) between 3.5 (for the protected meat with the film that incorporates 15% of PW) and 3.97 for the beef covered by the film that incorporates the shell. No significant differences were observed between them. 

The processing of the patties was carried out working in sterile conditions, which favors the initial contamination to be low (around 100 of CFU/g meat), and therefore the growth found is not very important.

There are reported cases where an antimicrobial agent is added to the gelatin film to extend the useful life of the beef meat from eight to 14 days (with respect to the microbial contamination and taking into consideration that it does not refer to ground meat, but to slices of meat) [81]. However, there are authors, like Bonilla Lagos et al. [82], who have studied samples with an initial contamination higher than 1000 CFU/g and after seven days some samples protected with film incorporating natural extracts reached levels between 5 and 7 (Log of CFU/g sample). However, it can be said that the films did not give significant protection against microbial contamination. 

Metmyoglobin was been analyzed. Myoglobin in fresh red meat can be found as deoxymyoglobin, oxymyoglobin and metmyoglobin. Deoxymyoglobin is responsible for the purple color in the meat. When exposed to oxygen it can be fast oxidized to bright red oxymyoglobin. Further oxygenation results in the formation of metmyoglobin, which is related to a loss of freshness in the meat and can be identified as a brown coloration.

**Figure 7:**
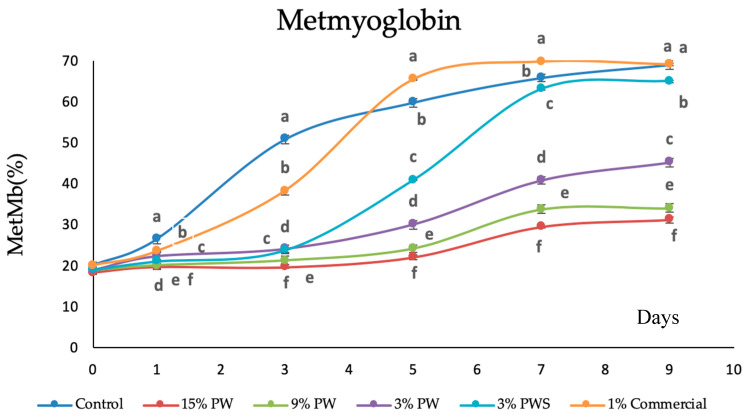
Metmyoglobin values of raw beef patties during storage at 4 ± 1 °C. Results represent the mean of three replicates (*n* = 3) and are expressed as mean value ± SD; different letters in the same day indicate significant difference between samples at *p* < 0.05, different capital letters indicate significant difference between storage days at *p* < 0.05 for the same sample. PW: Pecan walnut; PWS: Pecan walnut shell.

Figure 7 shows the myoglobin percentage of the different samples. After a week, all the samples were significantly different (*p* < 0.05) and two big groups could be distinguished. The first group is the control sample, the commercial 1% and the 3% PWS. They all present a metmyoglobin percentage higher than 60%, which correlates to the color shown by the different patties after a week of storage (a dark color, unpleasant at first sight).

In the other group are all the samples that incorporate films with PW, with a difference depending on the percentage of PW. The hamburger protected with the film that has more PW (15%) exhibits a lower metmyoglobin value, less than 30%, whereas the other two are between 34% and 41% for the film sample with 9% and 3%, respectively. 

This fact indicates that there is no clear correlation between the oxidation (measured through TBARs) and the color (measured by % of metmyoglobin). The samples covered with film containing PW show lower values of metmyoglobin, which could be due to some phenolic compounds that the PW contains. De la Rosa et al. identified, in pecan kernels, phenolic acids such ellagic acid, ellagic and gallic acid derivatives and monomeric flavan-3-ols. However, in the shells, they only identified ellagic and gallic acids [20]. These compounds on the kernel could slow down metmyoglobin formation. 

Several authors, like Bentayeb et al. found that surface metmyoglobin formation was depressed by active packaging containing rosemary, although the increase of rosemary concentration in the film did not cause significant differences [83]. 

## 4. Conclusions

The results showed that mechanical and water vapor barrier properties of the developed films varied depending on the concentration of the walnut, shell and synthetic antioxidant. With higher walnut concentration (15%), the permeability to water vapor was significantly lower than the control. Furthermore, with the addition of natural extracts and synthetic preservatives, the elongation at the break and Young’s modulus were lower than for the control. FTIR analysis allowed characterization of the molecular interactions occurring after the inclusion of pecan walnut and pecan walnut shell. The microstructural observations by SEM clearly demonstrated that the structure of the film containing walnut was heterogeneous, whereas the shell extract and commercial showed a homogeneous structure. Films with pure gelatin cannot act as an antioxidant shield to prevent food oxidation, but adding pecan walnut (15% concentration) improved stability of the stable DPPH radical by 30%. Furthermore, in the DSC, the addition of walnut (15 and 9% concentrations), allowed the formation of big crystals, which improved the thermal stability of gelatin films. The use of gelatin films with the incorporation of bioactive compounds coming from the walnut and the walnut shell have shown good protection against the oxidation of beef patties, which opens up a big field to the commercialization of meat products with lower quantities of synthetic products. However, the antimicrobial protection with the addition of walnut and walnut shell extracts was low.

## Figures and Tables

**Table 1:** Total Polyphenol Content (TPC) and radical scavenging values from the PW (pecan walnut) and PWS (pecan walnut shell).

Sample	TPC [mg GAE/g DW]	DPPH [µmol TE/g DW]
PW	20.55 ± 0.03 ^a^	37.63 ± 1.08 ^a^
PWS	72.96 ± 0.02 ^b^	205.12± 3.00 ^b^

^a,b^ Mean ± SD (*n* = 3). Different letter means within each column with different superscripts are significantly different (*p* < 0.05).

**Table 2:** FT-IR spectroscopy of gelatin film with PW (pecan walnut) and PWS (pecan walnut shell).

Peak (CM^−1^)	*n*.	Functional Group	Bond	Control Film	3% PW Film	9% PW Film	15% PW Film	3% PWS Film	1% Commercial Film	0.1% Commercial Film
3289.11–3281.09	1	Amines/Amides/-OH	N-H/O-H	81.09%	93.48%	93.59%	95.67%	--	88.07%	93.73%
3007.26–3007.59	2	Aromatic	C–H	--	--	93.90%	93.2%	--	--	--
2934.24–2923.27	3	Alquilo	–C–H	91.09%	91.84%	68.08%	71.25%	--	92.82%	95.14%
1743.74–1743.29	4	Acid carboxylic	C=O	--	95.32%	66.68%	72.24%	--	--	--
1630.86–1645.51	5	Carbonyl	C=O	62.12%	86%	89.37%	85.49%	98.37%	80.55%	71.14%
1548.37–1541.41	6	Amines blending	N–H	67.68%	87.51%	90.89%	88.05%	98.15%	83.08%	83.66%

**Table 3:** Thickness of the samples.

Sample	Thickness (mm)
Control	0.032 ± 0.01 ^d^
15% PW	0.150 ± 0.02 ^a^
9% PW	0.079 ± 0.00 ^b^
3% PW	0.047 ± 0.00 ^d^
3% PWS	0.076 ± 0.00 ^bc^
1% Commercial	0.051 ± 0.00 ^cd^
0.1% Commercial	0.047 ± 0.01 ^d^

^a,b,c,d^ Mean ± SD (*n* = 3). Different superscripts letter in the column means that are significantly different (*p* < 0.05). PW: Pecan walnut; PWS: Pecan walnut shell.

**Table 4:** Optical properties measurement.

Film	CIE L*	CIE a*	CIE b*	CIE ΔE
Control	93.75 ± 0.23 ^a^	−0.43 ± 0.34 ^de^	3.55 ± 0.43 ^e^	0.00 ± 0.00 ^e^
3% PW	91.72 ± 0.92 ^ab^	0.01 ± 0.16 ^de^	7.48 ± 1.82 ^cde^	4.78 ± 2.04 ^cde^
9% PW	89.20 ± 0.87 ^bcd^	0.33 ± 0.16 ^de^	12.77 ± 1.67 ^b^	10.32 ± 1.89 ^b^
15% PW	84.03 ± 0.60 ^e^	2.49 ± 0.35 ^ab^	18.51 ± 0.66 ^a^	18.08 ± 0.93 ^a^
3% PWS	86.71 ± 0.44 ^de^	3.19 ± 0.25 ^a^	12.21 ± 0.41 ^bc^	11.73 ± 0.65 ^b^
1% Commercial	93.61 ± 0.21 ^a^	–0.61 ± 0.23 ^e^	4.39 ± 0.45 ^de^	0.87 ± 0.32 ^de^
0.1% Commercial	93.62 ± 0.07 ^a^	–0.54 ± 0.02 ^e^	4.66 ± 0.12 ^e^	1.13 ± 0.13 ^e^

CIE L* represents the vertical coordinate of a three-dimensional arrangement of colors and has a value range from 0 (black) to 100 (White). CIE a* is the horizontal coordinate and a values range from –80 (Green) to +80 (red). CIE b* is the horizontal coordinate and the values go from –80 (blue) until +80 (yellow). The total difference in color is represented by CIE ΔE*. ^a,b,c,d^ Diverse letters in the same column indicate that there is a significant difference between them (*p* < 0.05)**.** PW: Pecan walnut; PWS: Pecan walnut shell.

**Table 5:** Effect of the addition of different concentrations of PW and PWS, and commercial preservative on Ultimate Tensile Strength (UTS), Elongation at Break (EB), Young’s Modulus (YM).

Sample	YM (MPa)	UTS (MPa)	EB (%)
Control	797.92 ± 250.90 ^ab^	39.93 ± 13.56 ^ab^	6.39 ± 0.12 ^d^
3% PW	615.47 ± 172.79 ^abc^	37.51 ± 2.70 ^ab^	8.68 ± 2.57 ^cd^
9% PW	358.96 ± 16.24 ^bcd^	16.74 ± 2.90 ^bc^	25.70 ± 1.99 ^b^
15% PW	96.70 ± 17.65 ^d^	5.95 ± 1.05 ^c^	31.19 ± 4.28 ^bc^
3% PWS	513.99 ± 34.01 ^abcd^	21.84 ± 1.83 ^abc^	6.39 ± 0.03 ^d^
1% Commercial	883.24 ± 16.84 ^a^	43.79 ± 3.68 ^a^	7.17 ± 1.39 ^d^
0.1% Commercial	964.25 ± 232.44 ^a^	48.06 ± 19.76 ^a^	8.29 ± 3.76 ^cd^

^a,b,c,d^ Mean ± SD (*n* = 3). Values that share the same superscript letter in each column are not significantly different (*p* < 0.05). PW: Pecan walnut; PWS: Pecan walnut shell.

## Abbreviations

PW, pecan walnut; PWS, pecan walnut shell; TPC, total polyphenols content; GAE, mg of gallic acid equivalents; DPPH, 2,2-diphenyl-1-picrylhydrazyl; Trolox, (6-hydroxy-2,5,7,8-tetramethylchroman-2-carboxylic acid); DW, dry weight; UTS, Ultimate tensile strength; EB, elongation at break; YM, Young’s modulus; ΔE*, total color differences value; XRD, X-ray diffraction; SEM, scanning electron microscopy; WVP, water vapor permeability; DSC, differential scanning calorimetry; DW, dry weight.
